# Comparative genomics of *Mycoplasma feriruminatoris*, a fast-growing pathogen of wild *Caprinae*

**DOI:** 10.1099/mgen.0.001112

**Published:** 2023-10-12

**Authors:** Vincent Baby, Chloé Ambroset, Patrice Gaurivaud, Laurent Falquet, Christophe Boury, Erwan Guichoux, Joerg Jores, Carole Lartigue, Florence Tardy, Pascal Sirand-Pugnet

**Affiliations:** ^1^​ Université de Bordeaux, INRAE, UMR BFP, F-33882, Villenave d’Ornon, France; ^2^​ Université de Lyon, Anses–Laboratoire de Lyon, VetAgro Sup, UMR Mycoplasmoses animales, 69007 Lyon, France; ^3^​ Department of Biology, University of Fribourg and Swiss Institute of Bioinformatics, CH-1700 Fribourg, Switzerland; ^4^​ Université de Bordeaux, INRAE, BIOGECO, 33610 Cestas, France; ^5^​ Institute of Veterinary Bacteriology, Vetsuisse Faculty, University of Bern, CH-3001 Bern, Switzerland; ^‡^​ Present address: CDVUM, Faculté de médecine vétérinaire, Université de Montréal, 3200 rue Sicotte, St-Hyacinthe, QC, J2S 2M2, Canada; ^§^​ Present address: Mycoplasmology, Bacteriology and Antibioresistance Unit, Laboratoire Anses Ploufragan Plouzané Niort, BP 53, 31 rue des fusillés, F-22440 Ploufragan, France

**Keywords:** *Mycoplasma feriruminatoris*, mollicutes, ruminant pathogen, *M. mycoides *cluster, mobile elements, polysaccharide biosynthesis, *oriC*

## Abstract

*
Mycoplasma feriruminatoris* is a fast-growing *Mycoplasma* species isolated from wild *Caprinae* and first described in 2013. *M. feriruminatoris* isolates have been associated with arthritis, kerato conjunctivitis, pneumonia and septicemia, but were also recovered from apparently healthy animals. To better understand what defines this species, we performed a genomic survey on 14 strains collected from free-ranging or zoo-housed animals between 1987 and 2017, mostly in Europe. The average chromosome size of the *M. feriruminatoris* strains was 1,040±0,024 kbp, with 24 % G+C and 852±31 CDS. The core genome and pan-genome of the *M. feriruminatoris* species contained 628 and 1312 protein families, respectively. The *M. feriruminatoris* strains displayed a relatively closed pan-genome, with many features and putative virulence factors shared with species from the *M. mycoides* cluster, including the MIB-MIP Ig cleavage system, a repertoire of DUF285 surface proteins and a complete biosynthetic pathway for galactan. *M. feriruminatoris* genomes were found to be mostly syntenic, although repertoires of mobile genetic elements, including Mycoplasma Integrative and Conjugative Elements, insertion sequences, and a single plasmid varied. Phylogenetic- and gene content analyses confirmed that *M. feriruminatoris* was closer to the *M. mycoides* cluster than to the ruminant species *M. yeatsii* and *M. putrefaciens *. Ancestral genome reconstruction showed that the emergence of the *M. feriruminatoris* species was associated with the gain of 17 gene families, some of which encode defence enzymes and surface proteins, and the loss of 25 others, some of which are involved in sugar transport and metabolism. This comparative study suggests that the *M. mycoides* cluster could be extended to include *M. feriruminatoris *. We also find evidence that the specific organization and structure of the DnaA boxes around the *oriC* of *M. feriruminatoris* may contribute to drive the remarkable fast growth of this minimal bacterium.

## Data Summary

The authors confirm all supporting data and protocols have been provided within the article or through supplementary data files (Figs S1–S5, available in the online version of this article, Tables S1–S3) available in the online version of this article. Assembled genomes and the plasmid generated for this study are available from GenBank (accession numbers CP113496, CP104008, CP113497, CP113498, CP104009, CP113499, CP113500, CP113495 and CP104010). The other genomes used in this study are listed in Tables 1 and S2 and are available in the GenBank database with the accession numbers specified in the tables.

**Impact Statement**

Mycoplasma are small bacteria involved in many diseases affecting humans and a wide diversity of animals. Ruminants of primary importance for agriculture and food supply are subject to mycoplasma diseases worldwide and ways to control them remain challenging. Studying mycoplasmas present in wildlife allows anticipating potentially emerging pathogens of livestock. This study provides the first whole-genome comparative overview of the *Mycoplasma feriruminatoris* species, a mycoplasma isolated in wild *Caprinae*. A set of 14 isolates collected between 1987 and 2017 were genome-sequenced and compared to get a picture of the diversity within the species and its relatedness to other ruminant mycoplasmas. The *M. feriruminatoris* strains displayed a relatively closed pan genome with some putative virulence factors, including the mycoplasma-specific MIB-MIP immunoglobulin cleavage system, a large repertoire of surface proteins and a complete biosynthetic pathway for galactan. Phylogenetic reconstructions and comparison of gene repertoires suggest that the related *M. mycoides* cluster, which comprises other ruminant pathogens, could be extended to include the *M. feriruminatoris* species. Besides its importance as a potential emerging pathogen, *M. feriruminatoris* has also attracted interest as a minimal bacterium with a noticeable fast growth (about 27–29 min to double population), which makes it a valuable candidate for designing bacterial chassis for biotechnological applications. The genetic basis for its fast growth characteristics has yet to be clarified but the specific organization of the genome around the replication origin could be part of it.

## Introduction

Within the prokaryotes, the class *Mollicutes* gathers bacteria characterized by their inability to synthesize peptidoglycan or the precursors necessary to build cell walls. They are consequently Gram-stain-negative, despite having evolved from Gram-positive bacteria, of which they constitute a distinct phylogenetic lineage. The class *Mollicutes* includes 11 genera [1], among which the *Mycoplasma* genus gathers the largest number of pathogenic or opportunistic species (*n*=78) and continues to grow as new species are regularly described in various hosts. Six new *Mycoplasma* species were described in the year 2022 alone [2–4]. *Mycoplasma spp*. genomes are small, with length varying from 580 to 1350 kbp, resulting in very limited metabolic pathways and thus fastidious growth that generally requires sterols and complex media. Their generation time varies widely but can exceed several hours for certain species. Their G+C content is low, varying from 23 % for *Mycoplasma capricolum* subsp. *capricolum* to 40 % for *M. pneumoniae *. They also share a specific pattern of codon usage with UGA encoding tryptophan.

The *Mycoplasma* genus is polyphyletic and can be divided into three distinct groups, i.e. the two clades Hominis and Pneumoniae, and a third one known as the clade Spiroplasma. This last one includes the ‘* M. mycoides* cluster’ that contains the type species of the genus despite its eccentric phylogenetic position [5]. The *M. mycoides* cluster evolved from insect-associated *Mollicutes* (*Spiroplasma, Entomoplasma* and *Mesoplasma *) to become ruminant pathogens capable of non-vectored direct transmission [6]. This evolution resulted from a combination of gene losses and more than 100 novel genes gained through horizontal gene transfer from donors potentially belonging to the Hominis/Pneumoniae lineages [7]. In particular, massive genetic exchanges have been predicted with the ruminant pathogen *M. agalactiae* from the Hominis clade [8]. Lo *et al.* [7] considered species of the *M. mycoides* cluster as hybrids ‘carved’ into shared ecological niches facilitating horizontal gene transfer.

The *M. mycoides* cluster – in its strict definition, which excludes some relatively close species such as *M. yeatsii* or *M. putrefaciens* – is an ecologically, phenotypically and genetically cohesive group of five major pathogenic ruminant (sub)species whose taxonomy was amended in 2009 despite conflict between phylogeny and taxonomy [9, 10]. The *M. mycoides* cluster includes four subspecies responsible for diseases listed by the World Organization for Animal Health (WOAH), namely *M. mycoides* subsp. *mycoides* (*Mmm*) and *M. capricolum* subsp. *capripneumoniae* (*Mccp*), which are the causative agents of contagious bovine and caprine pleuropneumonia, respectively, and *M. mycoides* subsp. *capri* (*Mmc*) and *M. capricolum* subsp. capricolum* (Mcap*), which are etiological agents of contagious agalactia. It also includes a fifth taxon pathogenic to cattle, *M. leachii *, which is a chimaera between *mycoides* and *capricolum* species that is seldom isolated [11–13]. The closely related species *M. feriruminatoris* was described 10 years ago [14] and later proposed to be part of the *M. mycoides* cluster in its enlarged definition [15].

Over time, isolates of *M. feriruminatoris* have been collected from wild *Caprinae*, i.e. the Alpine ibex (*Capra ibex*) or Rocky Mountain goat (*Oreamnos americanus*), either in the wild or in zoos [11, 14–16]. They all share rapid growth *in vitro*, with a generation time of 27–29 min at 37 °C [14, 17], remarkably shorter than the 80–200 min reported for members of the *M. mycoides* cluster [14, 18–21]. Their genetic diversity was originally thought to be low, when only five isolates, mainly from a German zoo, had been investigated [11], but was later shown to be higher once French isolates from ibex were investigated on top of the German isolates [15].

Despite a few dedicated papers and the availability of the genome of the type strain (G5847^T^), there are still gaps in knowledge of the *M. feriruminatoris* species. First, no specific virulence factors have been highlighted in the first genome announcement [17], even though *M. feriruminatoris* strains are genetically equipped to produce H_2_O_2_ [14], which is a potential virulence factor of mycoplasmas, that is controversially discussed [22–25]. Furthermore, *M. feriruminatoris* has been shown to produce one or two – depending on the isolate – types of capsular polysaccharides (galactan and/or β−1→6-glucan) [15], which is a true virulence factor for *M. mycoides* [26–28]. Second, questions remain about the level of divergence of *M. feriruminatoris* from members of the *M. mycoides* cluster in terms of gene content and genome organization. Third, the fast-growing capacity of *M. feriruminatoris* makes it an attractive species for the rational design of vaccine chassis [29], but the genetic bases for its fast growth are still unknown.

Here we used comparative genomics data to characterize the species *M. feriruminatoris *. The genomes of 14 *M *. *feriruminatoris* strains isolated over a 30 year period from 1987 until 2017, either from captive wild ruminants in zoos (*n*=5) or from free-roaming wild ruminants (*n*=9, mainly the French Alps) were compared to each other and to genomes from closely related species in terms of synteny, gene content, phylogeny, and specific features associated with virulence or host adaptation.

## Methods

### Strains, culture conditions and molecular biology methods

*M. feriruminatoris* strains were sourced from previous studies [11, 14, 16], from the Vigimyc network (strain F11561 [30]); and isolated from the diagnostic unit of the Institute of Veterinary Bacteriology at the University of Bern (isolate 14/OD_0492). Species assignment was verified using the species-specific PCR reported previously [15]. Only one other currently available genome, (isolate 14/OD_0535 [31], was not included in our work as it was released while most of our analyses were already completed.

All strains were cultured in PPLO medium supplemented as previously described [32] at 37 °C with 5 % CO_2_. Genomic DNA extraction was performed on cultures in mid-exponential phase using either 2 ml cultures for the phenol-chloroform method [33] or 10 ml cultures for the NucleoBond AXG column commercial kit (Macherey-Nagel).

### Genome sequencing, assembly and annotation

The full genome sequences of strains G5813/1+2, G1650, G1705, 8756–13 and 14/OD_0492 were obtained using PacBio sequencing technology, and deposited in GenBank under the accession numbers LR738858.1, LR739234.1, LR739233.1, LR739235.1 and LR739237.1, respectively. Whole-genome sequencing of *M. feriruminatoris* strains F11561, L13461, L14815, L14822, L15181, L15220, L15407 and L15568 was performed using a combination of Oxford Nanopore (ONT) and Illumina (paired-end 250 bp library) technologies (Table S1). The ONT reads were base-called using Guppy and demultiplexed using qcat (v.1.0.3, available at https://github.com/nanoporetech/qcat). The Illumina reads were trimmed using Trimmomatic (v.0.36, available at https://github.com/usadellab/Trimmomatic) [34], and the Illumina adapters, i.e. the first 5 bp and both ends, were removed using a 5 bp sliding window with a phred score of under 20. Quality of the Illumina reads was assessed before and after trimming using fastqc (v.0.11.5, available at https://www.bioinformatics.babraham.ac.uk/projects/fastqc). The ONT reads were filtered using filtlong (v.0.2.0, available at https://github.com/rrwick/Filtlong), and reads with a length <250 bp or sharing <87 % identity with the trimmed Illumina reads were excluded. The long reads were assembled using canu (v1.8, available at https://github.com/marbl/canu) [35] with an expected genome size of 1 Mbp.

The initial assembly was polished by iterative alignment of the trimmed Illumina reads using bwa-mem (v.0.7.15, available at https://github.com/lh3/bwa) [36] followed by correction using pilon (v.1.22, available at https://github.com/broadinstitute/pilon) [37]. The final manual polishing was done by iteratively performing a variant calling pipeline and correcting the variant positions in the assemblies between each iteration. The variant calling pipeline used bwa-mem to map the Illumina reads. GATK (v.3.7, available at https://github.com/broadinstitute/gatk/) IndelRealigner [38] was then used to perform local realignment around indels, read mate coordinates were added using the SAMtools (v.1.5, available at https://github.com/samtools/samtools) [39] fixmate command, duplicate reads were marked with the Picard toolkit (v.2.18.9, available at https://broadinstitute.github.io/picard), and finally the variant positions were called using GATK HaplotypeCaller with ploidy set to 1. The corrected genomes were then manually circularized and annotated using prokka (v.1.12, available at https://github.com/tseemann/prokka) [40] and genetic code no. 4. The completed genomes were submitted to GenBank (Table 1).

**Table 1.** *
M. feriruminatoris* strains used in this study

Strain name∗	Host	Year isolated	Location of isolation	Main clinical sign	Genome size (bp)	Genes	CDS	IS‡	MICE	Plasmid	Accession no.
8756–13	Rocky Mountain goat	<1987	USA	–	1 060 955	919	882	3	–	–	LR739235
G5847^T^	Ibex	1993	Berlin Zoo, Germany	arthritis	1 075 604	916	878	12	–	–	CP091032.1
G5813/1+2	Ibex	1993	Berlin Zoo, Germany	arthritis	1 037 206	919	882	13	–	–	LR738858
G1650	Ibex	1993	Berlin Zoo, Germany	arthritis	1 038 690	913	876	14	–	–	LR739234
G1705	Ibex	1993	Berlin Zoo, Germany	arthritis	1 038 707	915	878	14	–	–	LR739233
L13461	Ibex	2003	Savoie region, French Alps	septicemia	1 017 763	900	863	3	–	–	CP113496
L14822	Ibex	2007	Savoie region, French Alps	pneumonia	1 084 342	909	872	2	2	–	CP104008
L14815	Ibex	2007	Savoie region, French Alps	keratoconjunctivitis	1 021 626	863	826	2	–	–	CP113497
L15181	Ibex	2008	Savoie region, French Alps	keratoconjunctivitis	1 042 812	886	849	2	1	–	CP113498
L15220	Ibex	2009	Savoie region, French Alps	pneumonia	1 016 817	858	821	2	1	1†	CP104009
L15407	Ibex	2010	Savoie region, French Alps	pneumonia, keratoconjunctivitis	1 025 568	966	929	1	–	–	CP113499
L15568	Ibex	2011	Savoie region, French Alps	none (animal follow-up)	1 015 346	950	913	0	–	–	CP113500
14/OD_0492	Ibex	2014	Nature and Animal Park Goldau, Switzerland	liver, fibrinous polyarthritis	1 070 562	941	904	12	1	–	LR739237
F11561	Ibex	2017	Haute-Savoie region, French Alps	Unknown (animal found dead in the wild)	1 011 470	854	817	0	–	–	CP113495

*Origin of strains G1650, G5813/1+2, and G1705 are described in [14]; origin of strain 14/OD_0492 is reported in biosample record (SAMEA6237244).

†Plasmid accession number: CP104010.

‡These include IS1296 major and minor types.

### Comparative genomic analysis

Homologous protein sequence clustering was performed using get_homologues (v.05032019, available at https://github.com/eead-csic-compbio/get_homologues) [41] with the COGtriangle [42] clustering algorithm. Multiple sets of genomes were analysed (Table S2). One set included the *M. feriruminatoris* genomes only and was used to identify the core genome and pan-genome of this species. A second set included the *M. feriruminatoris* genomes as well as the *Mmc*GM12 genome (RefSeq accession no. NZ_CP001668) and was used to build the *M. feriruminatoris* phylogenetic tree. A third set included the *M. feriruminatoris* genomes, multiple complete genomes from members of the *M. mycoides* cluster, i.e. *Mmc* GM12 and 95 010 (RefSeq accession nos. NZ_CP001668 and NC_015431), *Mmm* Glasdysdale, PG1, Ben1, Ben50, Ben468, izsam_mm5713 and T1/44 (RefSeq accession nos. NC_021025, NC_005364, NZ_CP011260, NZ_CP011261, NZ_CP011264, NZ_CP010267 and NZ_CP014346), *Mcap* ATCC 27343 (RefSeq accession nos. NC_007633) *Mccp* 9231-Abomsa, 87 001, M1601, ILRI181 and F38 (RefSeq accession nos. NZ_LM995445, NZ_CP006959, NZ_CP017125, NZ_LN515399 and NZ_LN515398) and *M. leachii* PG50 (RefSeq accession no. NC_014751). This set also included other *Mycoplasma* strains, i.e. *M. putrefaciens* KS1, Mput9231 and NCTC10155 (RefSeQ accession no. NC_015946, NC_021083 and NZ_LS991954), *M. yeatsii* GM274B (RefSeq accession no. NZ_CP007520). Finally, the genome of *Mesoplasma florum* L1 (RefSeq accession no. NC_006055) was also used as an outgroup for construction of the phylogenetic tree.

In order to compare core and pan-genomes of *M. feriruminatoris* and related species, all the proteins of a combined set including the *M. mycoides* cluster members, *M. yeatsii *, *M. putrefaciens, Me. florum* and *M. feriruminatoris* were clustered based on sequence similarity. Three sets of genomes were compared, each with their own core and pan-genome reconstructed based on these clusters (Table S2). One set was formed with the 14 *M *. *feriruminatoris* strains, the second set was formed with the four *M *. *yeatsii* and *M. putrefaciens* strains, and the third set was formed with the 16 *M *. *mycoides *-cluster strains available in databases. *Me. florum* was excluded from the sets, but its proteins were used as an outgroup during the clustering process.

Synteny of the *M. feriruminatoris* genome was analysed by whole-genome alignment using Mauve (v.20150226, available at https://darlinglab.org/mauve/mauve.html) [43, 44]. Synteny of the single-copy core genes in all the *Mycoplasma* genomes was visualized using the GMV genome browser (v.1e-93, available at http://murasaki.dna.bio.keio.ac.jp/wiki/index.php?GMV) [45]. Proteins located in the putative *M. feriruminatoris* Mycoplasma Integrative Conjugative Elements (MICE) were compared to the proteins of ICEA_5632_-I from *M. agalactiae* 5632 and ICEM from *Mmc* GM12 [46] using blastp with default parameters using a 40 % protein identity cut off (v. 2.9.0, available at https://ftp.ncbi.nlm.nih.gov/blast/executables/blast+/LATEST/). Insertion sequence (IS) detection was performed using ISfinder (available at https://isfinder.biotoul.fr/) [47].

### DUF285 protein analysis

All predicted protein sequences in the *M. feriruminatoris* genomes were screened for DUF285 domains using CD-search [48]. Motif detection was then performed using MEME (v. 5.0.5, available at https://meme-suite.org/meme/) [49], initially with default parameters but then adjusting the motif length to 25 and 16 amino acids in two separate runs to refine the motifs. The motifs found in all the predicted proteins were then detected using mast (v. 5.0.5, available at https://meme-suite.org/meme/) [49] in its default parameters. Protein signal peptides were predicted using SignalP (v. 6.0, available at https://services.healthtech.dtu.dk/services/SignalP-6.0/) [50], and transmembrane domains were predicted using DeepTMHMM (available at https://dtu.biolib.com/DeepTMHMM) [51].

### *In silico* analysis of polysaccharide pathways

A tBlastX analysis was used with default parameters to retrieve putative enzymes involved in polysaccharide synthesis and described in *M. feriruminatoris* strain G5847^T^ [15] and in other mycoplasma species [27, 52]. All *M. feriruminatoris* genomes sequenced in this paper constituted the database and the galactan synthase MSC_0108 from *Mmm* PG1^T^(CAE76760.1) as well as GsmA from *M. agalactiae* 14 628 (EIN15433.1) were used as queries. Two hits were retrieved from the tblastX results. The typical synthase structures of the identified proteins were then confirmed by TMHMM2 showing four or seven transmembrane regions (available at https://services.healthtech.dtu.dk/services/TMHMM-2.0/) [53] and multiple alignment evidencing a glycosyltransferase specific cytoplasmic domain with the DXD and R/QXXRW-like motifs.

### Phylogenetic tree construction

**Fig. 1.**
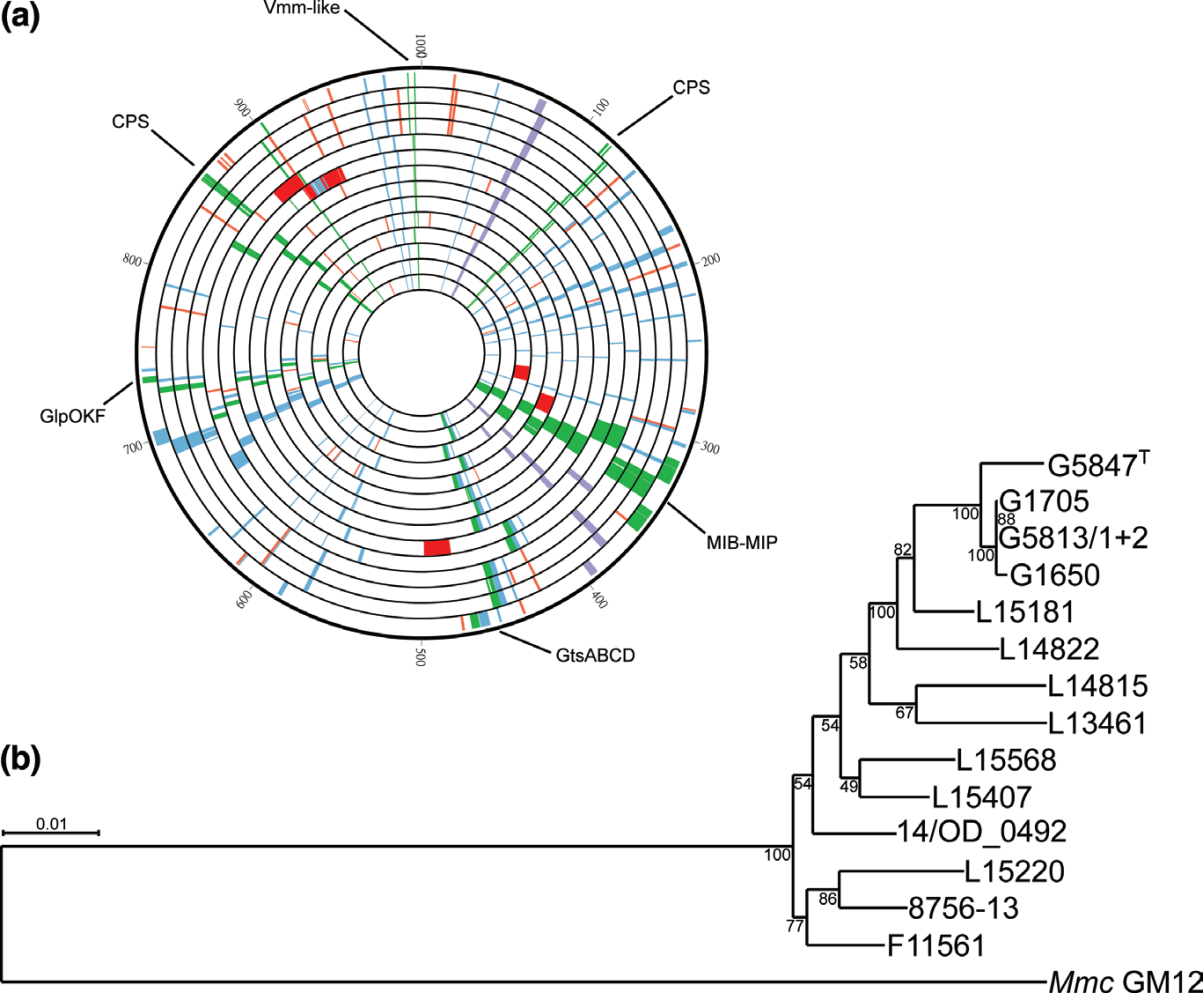
Genomic structure and phylogeny of the *M. feriruminatoris* genomes. (**a**) circular representation of all the *M. feriruminatoris* genomes, with size normalized to 1 Mbp. From outer to inner circles, the genomes are G5847^T^, G1650, G1705, G5813/1+2, L15181, L14822, L14815, L15407, L15568, 14/OD0492, L15220, 8756-C13, F11561 and L13461. Genes are coloured based on function, with virulence genes in green, insertion sequences (IS) in orange, rRNAs in purple, DUF285 proteins in blue, and MICE genes in red. (**b**) Phylogenetic tree of the *M. feriruminatoris* strains. The tree was constructed using the maximum-likelihood method and inferred from the concatenated alignments of 555 single-copy core protein sequences. The alignment matrix contained 186 920 amino acid sites. *Mmc* strain GM12 was used as the outgroup, and 450 bootstrap replicates were run. Bootstrap values are shown as node labels.

The phylogenetic trees were constructed using, respectively, 555 single-copy core genes for the intra-species tree (Fig. 1b) and 294 single-copy core genes for the tree including other strains for the *M. mycoides* cluster (Fig. 5). For each set, the protein sequences were aligned using Clustal Omega (v.1.2.1, available at http://www.clustal.org/omega/) [54] and the alignments were then concatenated. Unaligned and low-confidence regions were removed from the alignment using Gblocks (v.0.91b, available at molevol.cmima.csic.es/castresana/Gblocks.html) [55], thus producing sequence matrices of 186 920 and 92 785 amino acid sites for the *M. feriruminatoris* tree and the *M. mycoides* tree, respectively. The evolution model for tree construction was determined using ProtTest (v.3.4.2, available at https://github.com/ddarriba/prottest3) [56], and in both cases the CpREV model [57] was identified as the best model. The trees were then created with RaxML (v.8.2.12, available at https://github.com/stamatak/standard-RAxML) [58] using the GAMMA model of rate of heterogeneity, and 450 and 150 bootstrap replicates were made for the *M. feriruminatoris* tree and the *M. mycoides* tree, respectively, using the autoFCbootstopping criterion to determine the number of replicates.

### Ancestral genome reconstruction

The evolution of the gene-family contents over the course of the evolution of *Mycoplasma* species included in this work was studied using the COUNT software (available at http://www.iro.umontreal.ca/~csuros/gene_content/count.html) [59]. We used the phylogenetic tree described above with 14 *M *. *feriruminatoris* strains, 16 *M. mycoides* cluster-related strains, three *M *. *putrefaciens* strains, one *M *. *yeatsii* strain and the outgroup *Me. florum *, and we used the presence/absence matrix produced in the comparative analysis to monitor the occurrence of 2615 gene families. We used a birth-and-death model to calculate the posterior probabilities, and we used a gain–loss model with a Poisson distribution at the root and set the edge length, loss and gain rates at four gamma categories to maximize the likelihood of the optimized model.

## Results and discussion

### A homogeneous species with a closed pan-genome

In order to get an overview of the *M. feriruminatoris* species and further investigate its evolutionary relationship with other species from the *M. mycoides* cluster, we sequenced the genome of 13 strains isolated from different regions and years and from animals showing different clinical signs (Table 1). We used a combination of Illumina short reads and PacBio or ONT long reads to produce complete circular chromosome sequences for all the strains. Strain G5847^T^ was included in the comparative genomic analysis. The average chromosome size of the 14 strains was 1,040±24 kbp with the smallest and largest genome at 1 011 470 bp and 1 084 342 bp for strains F11561 and L14822, respectively (Table 1). As expected for members of the class *Mollicutes *, the G+C content of the genomes was low, at an average of 24.24±0.04 %. The genomes were annotated using prokka [40] that predicted between 853 and 941 (average 890) genes per genome, including 816 to 904 (average 852) protein-encoding genes (Table 1). Each genome had two rRNA loci encoding the 5S, 16S and 23S rRNAs separated by approximately 318 kbp and located on the same half of the genome relative to the chromosomal origin of replication and the terminus (Fig. 1a). A total of 30 tRNAs genes and a single tmRNA were predicted in every genome.

**Fig. 2.**
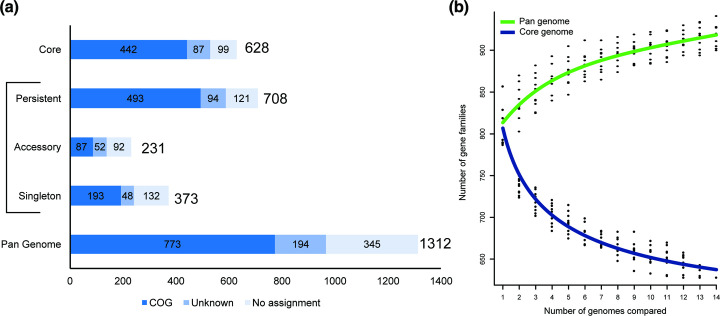
Core and pan-genomes of the *M. feriruminatoris* species. (**a**). The pan-genome is composed of 1312 gene families encoding proteins, of which 373 are singletons (present in a single genome), 708 are persistent (present in at least 13/14 genomes), 628 are core (present in all genomes), and the remainder (*n*=301) are accessory (present in 2 to 12 genomes). The sum of persistent, accessory and singleton genes constitute the total pan-genome genes. Gene families with predicted eggNOG (NOG) function categories are indicated as follows: dark blue, known; medium blue, unknown; light blue, no assignment. (**b**). Gene number estimation curves for the *M. feriruminatoris* core genome (blue, bottom curve) and pan-genomes (green, top curve) were generated using the methods described in Willenbrock *et al*. [73] and Tettelin *et al.* [74], respectively.

A total of 11 935 proteins were predicted from the 14 *M *. *feriruminatoris* genomes. A phylogenetic tree was built using the sequence of 555 single-copy core proteins found in all *M. feriruminatoris* strains and in *Mmc* strain GM12, which was used as the outgroup (Fig. 1b). In the resulting tree, *M. feriruminatoris* strains were grouped into a homogeneous single branch at a short distance from the *Mmc* root. Four strains, i.e. G5847^T^, G1650, G1705 and G5812/1+2, that had been isolated in a narrow time-window (1993–1994) from ibexes that were hosted in Berlin zoo and showed similar clinical signs, were very closely related, as expected. However, besides these zoo strains, we found no further correlation between phylogenetic branches and location or time of isolation. For example, the most-recently isolated strain F11561 (2017) and the least-recently isolated strain 8756–13 (1987), which were also isolated on two separate continents (Europe and North America, respectively), were found in the same branch. The core and pan-genomes of the *M. feriruminatoris* species were determined based on the 11 390 proteins grouped in 1312 clusters of homologs retrieved from the 14 *M *. *feriruminatoris* genomes (Fig. 2). A core set of 628 clusters of protein-encoding genes was present in every genome, representing an average of 47.9 % of all clusters within a given strain. Considering possible sequencing errors, this value was extended to 708 clusters of persistent gene predicted from at least 13 out of the 14 genomes. A total of 373 singleton protein clusters representing 28.4 % of all clusters were present in only one strain. The *M. feriruminatoris* strains had an average of 27±17 singleton clusters representing 3.2 % of their total number of different clusters, but most of them contained short protein sequences measuring less than 200 amino acids, which suggests that many could be annotation artefacts (i.e. pseudogene remnants). These potential artefacts in strain-specific proteins together with the slow rise of the pan-genome estimation curve (Fig. 2b) suggest that the *M. feriruminatoris* species possesses a closed pan-genome with most gene families shared between multiple strains, which is consistent with the recently proposed genomic definition of a bacterial species [60]. Nonetheless, as our isolates come primarily from two locations in Europe, except the old historical strain from the USA, any new sequences of *M. feriruminatoris* from other parts of the world would be welcome to confirm this overall evolution of the species.

A detailed analysis of the distribution of gene families from the core, persistent, accessory and singleton genomes into functional categories was then undertaken (Fig. 2a). Among the 1312 gene families of the *M. feriruminatoris* pan-genome, 773 (58.9 %) were assigned to non-supervised orthologous groups (eggNOG) with one or several functional categories, whereas 194 (14.8 %) were assigned to an eggNOG with unknown functional category, and 345 (26.3 %) were not assigned. The proportions of clusters assigned to an eggNOG with a known function varied between approximately 70 % in the core or persistent genome and 38 % in the accessory genome. Although the eggNOG domain of ‘genetic information storage and processing’ categories appeared to be highly represented in core, persistent, accessory and singleton genomes, further analysis revealed significant differences (Fig. S1). Although the persistent genome encompassed 81.8 % (126/154) and 59.3 % (32/54) of gene families from categories J (translation, ribosomal structure and biogenesis) and K (transcription), it only encompassed 35.5 % (54/152) of gene families for category L (replication, recombination and repair), whereas the singleton genome encompassed 41.5 % (63/152) of category-L gene families, which suggests fast turnover of some genes. Further analysis indicated that many of these highly volatile genes could be associated with mobile genetic elements (MGE) such as IS or MICEs. The 156 gene families involved in cellular processes and signalling represented 20.2 % of the gene families with known eggNOG function categories, the most represented category being V (defence mechanism) with (62 %, 16/26) and (48 %, 22/46) families present in accessory and singleton genomes, respectively, which also suggests a fast evolution of the corresponding repertoire of genes among *M. feriruminatoris* strains. In contrast, this category represented only 19 %(16/84) of the gene families involved in cellular processes and signalling in the persistent genome. Taken together, our findings from detailed analysis of the *M. feriruminatoris* pan-genome point to a global conservation of gene families involved in information processing and central metabolism and more strain-specific repertoires associated with MGEs and defence mechanisms.

### A highly syntenic structure, locally influenced by the mobilome

**Fig. 3.**
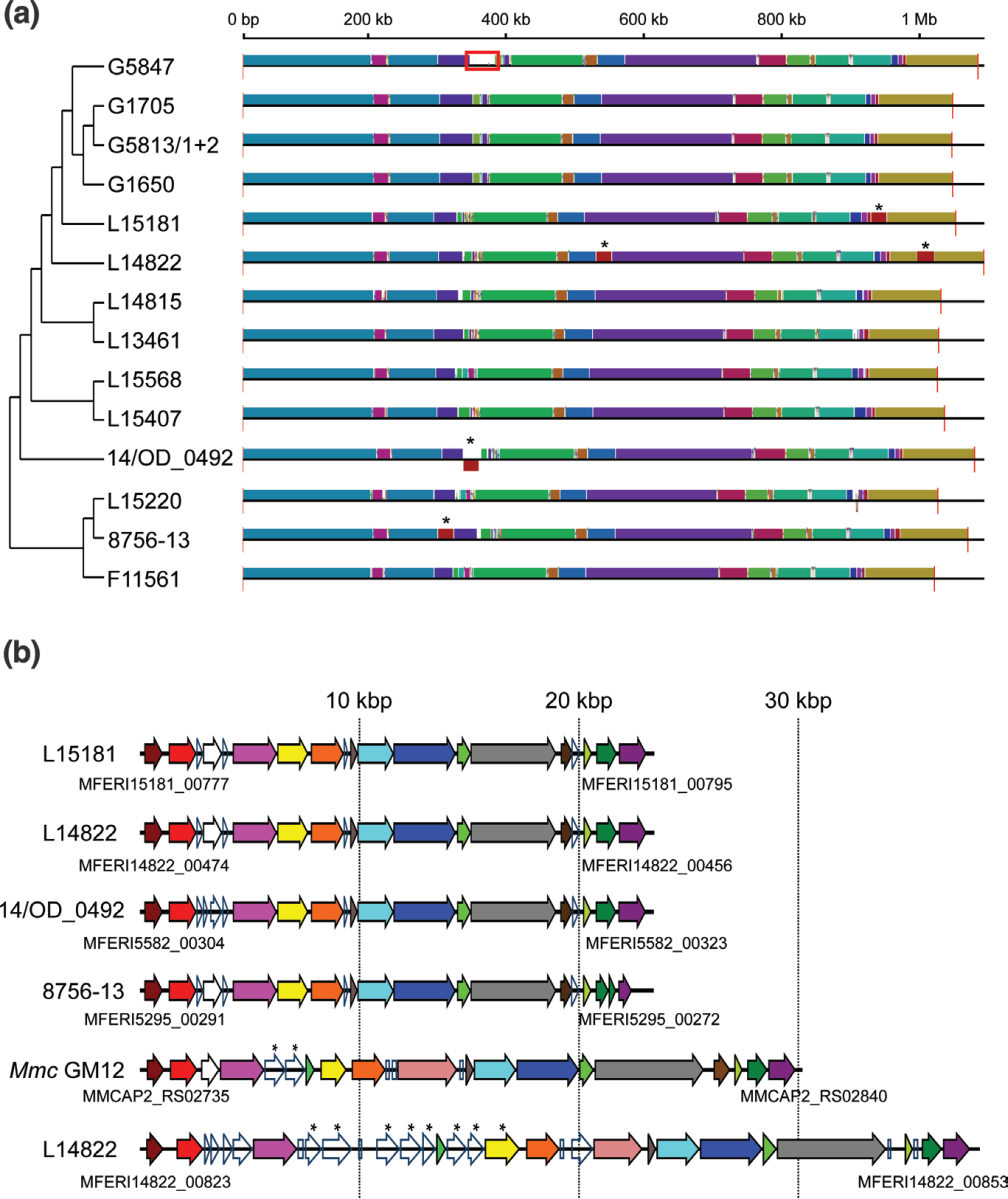
Synteny between the *M. feriruminatoris* genomes. (**a**) Conserved synteny blocks shared by the different strains. The tree topology represented on the left side corresponds to the tree in Fig. 1b. MICE locations are identified with an asterisk, and the 35 kbp duplication in strain G5847T is framed in red. (**b**) *M. feriruminatoris* MICE structure. Homologous genes between the *M. feriruminatoris* and *Mmc* GM12 MICEs are represented with the same colour. The MICE strain of origin is indicated on the left of each MICE, and the locus tags of the first and last genes of the MICEs are specified under their respective track.

Synteny blocks from the 14 *M *. *feriruminatoris* genomes shared mostly the same order (Fig. 3a). There was no observable major reorganization except one noticeable ~35 kbp duplication event in the G5847^T^ genome [29], resulting in six copies of the immunoglobulin cleavage proteins MIB and MIP [61] while the other strains had either three or four copies.

A synteny block of ~23 kbp found in strains L15181, L14822, 14/OD_0492 and L15220 was shown to be located in different regions and orientations depending on the strain (Fig. 3a, red block tagged with an asterisk). Upon closer inspection, these loci were shown to be akin to the MICE identified in *Mmc* GM12 (Fig. 3b). Almost all the *Mmc* GM12 MICE genes had a homologue in the *M. feriruminatoris* MICEs and were also in the same order. A second putative MICE was identified in strain L14822, although with a slightly different structure and encoding multiple proteins within the DUF285 domain.

With the exception of strains L15568 and F11561 that had none, all *M. feriruminatoris* strains presented variable numbers of IS highly related to IS1296 [62] of the IS3 superfamily found in *M. mycoides *-cluster mycoplasmas (Table 1). Two different IS1296-like elements were identified, the most frequently detected had a ~86 % nucleotide identity (major IS1296-like) with the original IS1296 sequence and a second type with >95 % identity (minor IS1296-like). Most strains had up to three complete copies of the major type, while some, notably those from zoo isolates, had 10 to 14 copies. The minor type was detected in two copies in both L13461 and 14 OD_0492 strains. The surge of ISs observed in these strains did not modify the genome organization, which contrasts with large inversions evidenced in the genomes of some *M. mycoides *-cluster strains such as *Mmc*95010 [63], PG3 and 152/93 [64] and *Mmm* T1/44 [65]. We found no traces of prophages or CRISPR loci in *M. feriruminatoris* genomes.

Only one out of the 14 strains (L15220) was shown to harbour a 3 301 bp plasmid. The pL15220 plasmid was longer than most of the plasmids described so far within the *M. mycoides* cluster but close to the size of pMyBK1 plasmid from *M. yeatsii* (3 432 bp) [66], and had a G+C content of 26.5 %. The strongest pairwise nucleotide identity with mycoplasma plasmids was obtained with plasmid pMmc-95010 of *M. mycoides* subsp. *capri* str. 95 010(1 850 pb and 48.6 % of identity) [63], despite a noticeable difference in size of circa 1400 bp. The pL15220 plasmid encodes two CDSs, including a 649 aa hypothetical protein (HP; with a partial homology to COG3378) and a predicted CopG-family transcriptional regulator whose gene shares 82 % nucleotide identity with its homologue on pMmc-95010 (Fig. S2). Noticeably, three regions of 94 to 293 nt shared 76–100% nucleotide identity with three different regions of the L14822-specific MICE, a feature already described in work comparing the plasmid and MICE of the *Mmc* 95 010 strain [63] and that suggests genetic exchanges among these MGEs. The pL15220 plasmid does not encode a Rep2 protein with a pfam01719 domain shared by most replicases [66]. This finding is consistent with the absence of a double-strand origin (dso) where the Rep proteins normally cleave [at a conserved site TACTAC(C)G/A] the positive DNA strand to start the rolling-circle replication [67]. In contrast, a sso block (lagging-strand initiation site) similar to pMmc-95010 was identified next to the gene encoding the HP mentioned above. Its mosaic structure together with the presence of a CDS encoding a hypothetical protein with no homologues in databases suggests that the pL15220 plasmid may belong to a new plasmid family whose emergence was marked by recombination events with other mycoplasma plasmids and MICEs.

Because of its overall highly conserved genome synteny and its coherent positioning within the intraspecies phylogeny tree, *M. feriruminatoris* G5847^T^ might be considered a valuable representative of the species. In many cases, the very first strain chosen for genome sequencing is a lab strain that has encountered an unknown number of passages and whose origin often remains unclear. This was the case for the *Mmm* PG1^T^ genome sequenced in 2004 whereas the isolate dated back to 1931 [68]. Soon after, PG1^T^ was shown to differ greatly from other field strains with a notable 24 kb genetic locus repetition and hence was not the best representative of this important bovine pathogen [69]. In the case of *M. feriruminatoris *, the G5847^T^ isolate also has one major, near-perfect 35 kbp duplication that was not present in other genomes. This duplication was ascertained using two independent long-read sequencing strategies [29], and we concluded it may have occurred during passages in the lab.

### A variable repertoire of enzymes involved in polysaccharides' biosynthesis

**Fig. 4.**
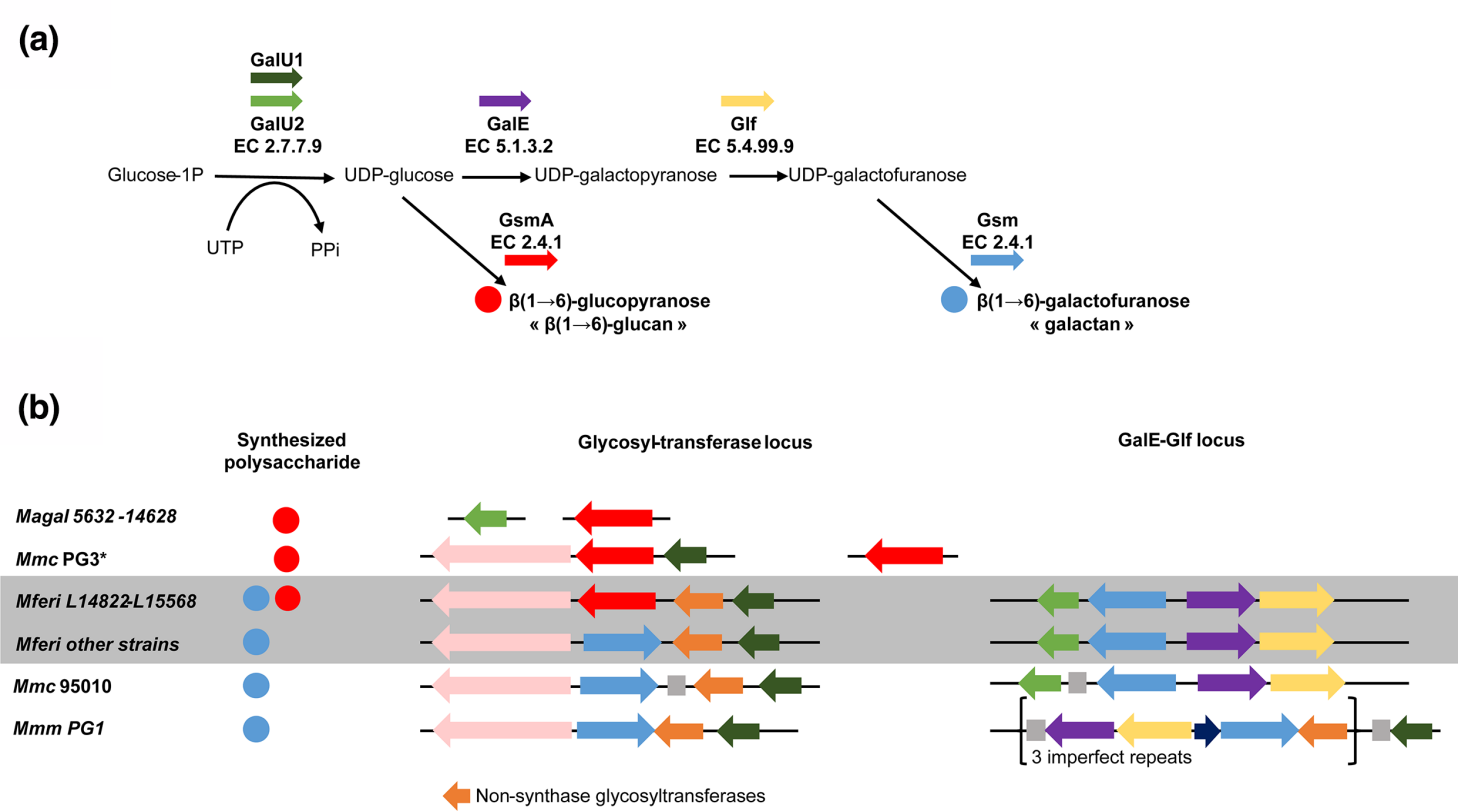
Polysaccharide biosynthesis pathways. (**a**) Schematic representation of metabolic pathways involved in β(1→6)-glucan and galactan synthesis. Glucose-1P is transformed into UDP-glucose by a glucose-1-phosphate uridylyltransferase (GalU1 or GalU2). An UDP-glucose 4-epimerase (GalE) and an UDP-galactofuranose mutase (Glf) successively transform the UDP-glucose into UDP-galactofuranose. Finally, one (or several) glycosyltransferases (GT) with synthase activity (Glycan Synthase of Mollicute, Gsm or GsmA for *M. agalactiae *) builds and exports the final polysaccharide, a polymer of galactofuranose, the galactan (blue circle) or β(1→6)-glucan (red circle). (**b**) Organization of the genes encoding the corresponding enzymes amongst genomes of the *M. mycoides *-cluster strains and *M. agalactiae* (*M. agal*). Red or blue circles indicate the nature of the polysaccharide [as in (a)], coloured arrows represent coding sequences [synthases are in blue or red as in (a), other glycosyltransferases lacking the structural signature of synthases are in orange, and dark blue and pink arrows represent hypothetical proteins and peptidases, respectively]. Grey squares represent transposases.

*M. feriruminatoris* isolates were previously shown to produce a capsule composed of either galactan and/or β-(1→6)-glucan depending on the isolates [15]. We used tBlastX to search for homologues of enzymes involved in polysaccharide biosynthesis, as predicted in *M. feriruminatoris* G5847^T^ and in other *Mycoplasma* species [15, 27, 52]. A complete putative biosynthetic pathway for galactan was predicted in all *M. feriruminatoris* isolates, with genes clustered in two different genomic locations (Figs 1 and 4).

Eleven out of the thirteen newly sequenced strains carried two nearly identical *Gsm* genes encoding genuine galactan synthases that were homologues to the synthase encoded by MSC_108 in *Mmm* PG1^T^ [27]and carry the typical four transmembrane domains (TMDs) and a cytoplasmic domain with DAD and QRMRW motifs. One is located in the glycosyl-transferase locus, and the other is located in the GalE-Glf locus (Fig. 4b). In contrast, but in agreement with previous PCR findings [15], isolates L14822 and L15568 have one *Gsm* copy only. However, these two isolates harbour a different synthase, at the exact same position as the first *Gsm* in other strains, with seven TMDs and a cytoplasmic domain harbouring DXD and RXXRW motifs, homologous to the GsmA synthase involved in β-(1→6)-glucan production in *M. agalactiae* 14 628 and *Mmc* PG3 [27, 70]. The GsmA protein of *M. feriruminatoris*is closer to its homologue in *Mmc* PG3 than in *M. agalactiae* 14628 (81.2 % vs 66.5 % protein identity). The *GsmA* proteins of isolates L14822 and L15568 are 99.9 % identical. This ‘surgical’ genomic ‘replacement’ of a *Gsm* gene by a *GsmA* gene in two isolates only and the mechanism beyond the replacement are an intriguing feature of the otherwise very syntenic clusters for polysaccharide biosynthesis.

### A large repertoire of lipoproteins and proteins with DUF285 domains

Using SignalP, we found 1104 lipoprotein signal peptides in the 14 *M *. *feriruminatoris* genomes corresponding to an average of 79 [77–83] lipoproteins per strain. blastP analyses confirmed the presence of the usual main immunogenic lipoprotein repertoires of the *M. mycoides* cluster, including LppA, LppB, LppC, LppQ and the variable surface protein Vmm. In each genome, *lppB*, *lppC* or *lppQ* genes were present in a unique copy, whereas up to four genes encoding *lppA* and six to seven Vmm copies were detected per genome. Interestingly, isolates collected from wild ibex had three to four copies of the *lppA* gene, with either two pairs of adjacent genes in different regions or one pair and a singleton, whereas isolates from zoos harboured one to three copies of the gene and several (up to four) truncated genes. The proximity of IS could explain the presence of duplicated, truncated LppA-encoding genes.

Lipoproteins with multiple DUF285 domains were also detected in all *M. feriruminatoris* genomes (Fig. S3). DUF285 domains are presented as one of the top ten relevant domains for animal host classification [71]. Proteins with DUF285 domains, of as-yet unknown function, are also found in the members of the *M. mycoides* cluster [8] and in other ruminant mycoplasmas including *M. agalactiae* and *M. bovis *. DUF285-domain proteins contain varying numbers of 25 amino acid long-tandem repeats [72]. The tandem repeats of the *M. feriruminatoris* DUF285 proteins are also preceded by a 16-residue motif of 12 to 14 amino acids upstream of the first repeat (Fig. S3A). This upstream motif is found for almost every DUF285 protein, the only exceptions being proteins <200 amino acids long (Fig. S3B). Furthermore, this upstream motif was only found in DUF285 proteins. A total of 380 DUF285 proteins were found in *M. feriruminatoris* genomes, i.e. an average of 27 DUF285 domain proteins per strain. The DUF285 proteins grouped into 51 clusters based on the comparative genomic analysis, and 11 of them were part of the core genome. Two of the core DUF285 protein clusters are also duplicated between two to five times in every strain. The DUF285 protein clusters present in at least ten strains are mostly predicted to also possess a signal peptide or a lipoprotein signal peptide, but some are also predicted to have transmembrane domains at either the C or N terminus. When more than one transmembrane domain was predicted, they were located at both the C and N terminus of the proteins. Remarkably, eight CDS encoding DUF285 proteins were all found in the L14822-specific MICE (Fig. S2).

### *M. feriruminatoris*is much closer to any member of the *M. mycoides* cluster than to either *M. putrefaciens* or *M. yeatsii *.

**Fig. 5.**
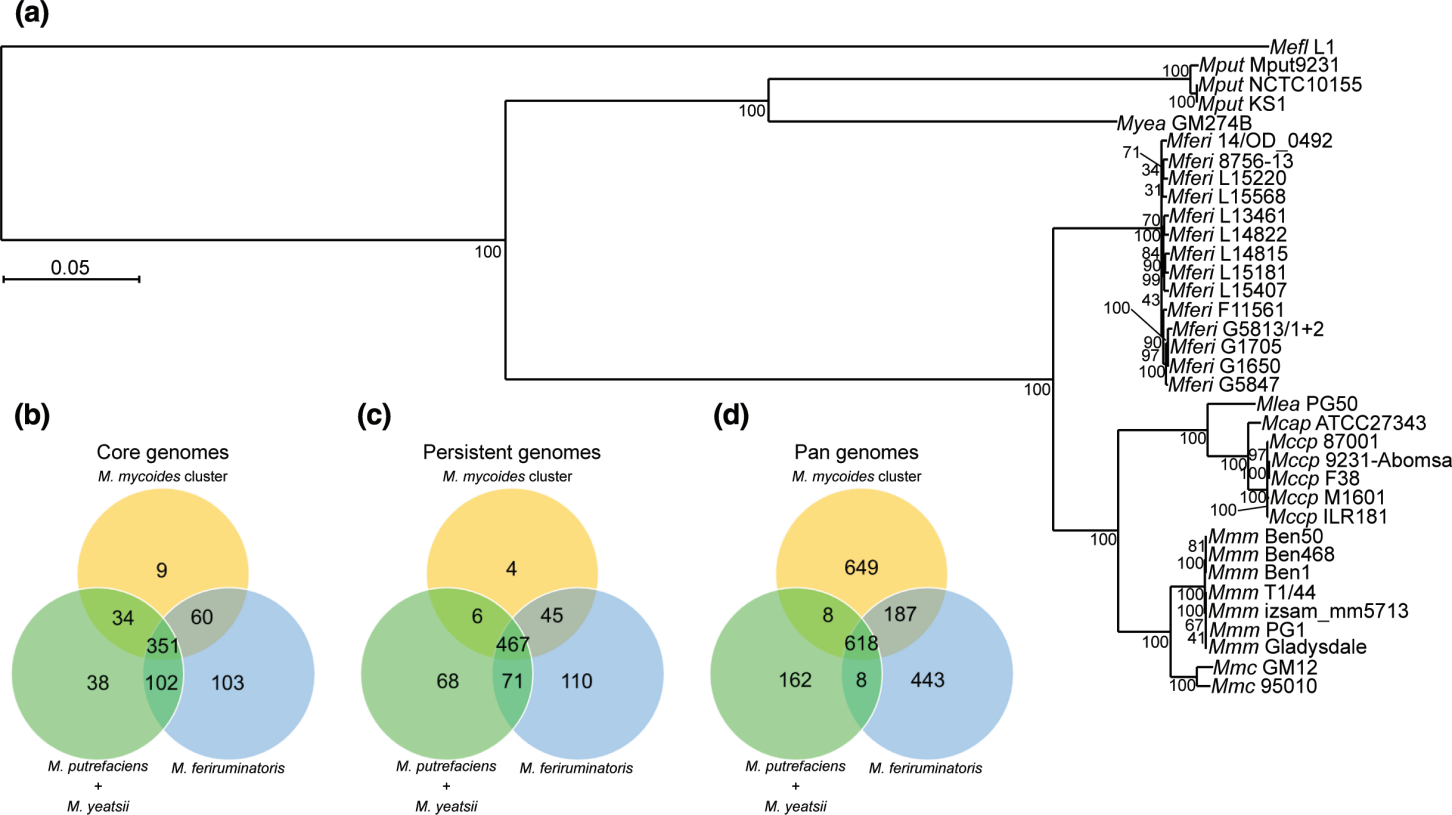
Comparison between *M. feriruminatoris *, the *M. mycoides* cluster, and their closest relatives. (**a**) A phylogenetic tree inferred using the maximum-likelihood method from the concatenated alignments of 294 single-copy core protein sequences, resulting in a total of 92 785 amino acid sites in the alignment matrix. *Me. florum* L1 was used as the outgroup, and 150 bootstrap replicates were run. Bootstrap values are shown as node labels. Venn diagrams of the protein clusters from the (**b**) core genome, (**c**) persistent genome, and (**d**) pan-genomes shared between *M. feriruminatoris,* the *M. mycoides* cluster, and their closest relatives.

To evaluate the relatedness and specificities of *M. feriruminatoris* compared to mycoplasmas from the *M. mycoides* cluster – in its strict definition – and closely-related ruminant species (i.e. *M. yeatsii* and *M. putrefaciens *), we ran a global comparative genomics analysis including 21 genomes in addition to the 14 from *M. feriruminatoris* (Table S2). In a high-resolution phylogenetic reconstruction based on 294 single-copy core proteins, *M. feriruminatoris* appeared much closer to any of the *M. mycoides* cluster members than to *M. putrefaciens* or *M. yeatsii* (Fig. 5a). This result is in accordance with previous phylogenetic studies based on 16S rRNA gene sequences [14] and the single protein FusA [15]. The monophyletic clustering of all *M. feriruminatoris* strains is also in agreement with the definition of a homogeneous species. The global phylogeny therefore indicates that *M. feriruminatoris* is more closely related to the *M. mycoides* cluster than to *M. putrefaciens* and *M. yeatsii *.

### What makes the difference between *M. feriruminatoris* and related species?

To identify specificities of the *M. feriruminatoris* species compared to phylogenetically related ruminant species, core and pan-genomes were constructed for three sets of genomes including 14 *M *. *feriruminatoris* strains, 16 *M *. *mycoides *-cluster strains and 4 *M *. *yeatsii* and *M. putrefaciens* strains, respectively. The core genomes of each set were similar in size, with 616 clusters for *M. feriruminatoris *, 454 for the *M.mycoides*-cluster strains, and 525 for *M. putrefaciens* and *M. yeatsii *. We determined the overlap between these core genomes (Fig. 5b) and found that 351 protein clusters were shared by all three core genomes. The *M. feriruminatoris* core genome intersects with a very similar number of genes to the other two, sharing 453 protein clusters with the *M. putrefaciens* and *M. yeatsii* core and 411 with the *M. mycoides *-cluster core. The number of shared protein clusters was extended to 467 when considering the persistent genomes instead of the strict core genomes (Fig. 5c). We also compared the pan-genomes of the three sets (Fig. 5d). The relatively small pan-genome for the *M. putrefaciens* and *M. yeatsii* set (796 protein clusters) might be explained by both their small genome sizes and the limited number of genomes available. In contrast, the pan-genomes of the two other sets were larger, with 1256 clusters for *M. feriruminatoris* and 1462 for the *M. mycoides* cluster. As these last two sets were composed of a similar number of ~1 Mbp genomes (Table S2), this result suggests that the gene diversity within the *M. feriruminatoris* species was comparable to the gene diversity of the whole *M. mycoides* cluster. A total of 618 protein clusters were present in at least one strain of the three sets, which represents 49.2, 42.3 and 77.6 % of the *M. feriruminatoris* pan-genome, *M. mycoides *-cluster pan-genome, and *M. putrefaciens–M. yeatsii* pan-genome, respectively. The *M. putrefaciens–M. yeatsii* pan-genome barely intersects with the other two sets, sharing only eight protein clusters with each, which further illustrates the distance between this group and the other two groups that overlap with 187 protein clusters.

To document the evolution of gene repertoires during the speciation process of *M. mycoides* cluster-related ruminant mycoplasmas, we employed an ancestral genome reconstruction approach using the birth-and-death model implemented in COUNT [59]. Starting from the content of the protein clusters and the phylogenetic relationship of included genomes, ancestral genome contents were simulated at each node of the phylogenetic tree with posterior probabilities for the protein cluster sizes (Fig. S4). We thus produced aggregate information for each inner node (number of clusters present with posterior probabilities superior to 0.5) and for each edge leading to the node (cluster gains, cluster losses). The last common ancestor (LCA) of *M. feriruminatoris* was proposed to include 805 protein clusters (node 16), which is close to the average calculated from the 14 *M *. *feriruminatoris* genomes with 786 protein clusters. Evolution from the *M. feriruminatoris */* M. mycoides* LCA (node 32) to the *M. feriruminatoris* LCA (node 16) was associated with the gain of 17 protein clusters and the loss of 25 protein clusters (Table S3). Among the 17 gained clusters, 7(41.2 %) are specific to the *M. feriruminatoris* core genome and totally absent from species belonging to the *M. mycoides* cluster. Further investigation indicated that three clusters might be involved in bacterial defence mechanisms, and four were predicted as surface proteins.

The 25 lost protein clusters included eight involved in metabolism (mainly sugar transport and metabolism), seven involved in information storage and processing (i.e. restriction modification systems and MGE), and one involved in cellular processes and signalling. Note that some of the predicted losses during *M. feriruminatoris* speciation were also predicted to have happened during the evolution of other caprine mycoplasmas. This was the case for genes involved in trehalose metabolism and for the MurR-RpiR family transcription regulator that were also lost in the *M. putrefaciens/M. yeatsii* LCA and *Mcap/Mccp* LCA but maintained in the *M. leachii* LCA and the *Mmm/Mmc* LCA. The main nodes leading to species (nodes 16, 21, 30) and subspecies (nodes 20 and 28) clearly show that these evolution steps were marked by gains of genes mainly associated with defence systems and surface proteins and by losses of genes involved in various aspects of cellular life, notably carbohydrate metabolism (Table S3). These differences in gene categories involved in gains and losses during the emergence of *M. feriruminatoris* suggest that the speciation process might be associated with key metabolic changes and with developments of elements (surface proteins) involved in host–pathogen interaction.

One of the main traits of the *M. feriruminatoris* species is its fast-growing capacity in axenic media, with a reported doubling time of ~30 min at 37 °C [14]. In comparison, members of the *M. mycoides* cluster have generation times ranging from 80 to 200 min [14, 18–21]. Our comparative analyses did not identify specific metabolic pathways that could explain this noticeable difference. In order to further investigate the genetic basis of the fast-growing *M. feriruminatoris* phenotype, we compared the genomic regions encompassing the predicted replication origins of the chromosomes of *M. feriruminatoris* strains and related species (Fig. S5). All *M. feriruminatoris oriC* regions were highly similar, with intergenic sequences located upstream and downstream of the *dnaA* gene containing seven and one predicted DnaA boxes, respectively. Interestingly, upstream of the *dnaA* gene, one DnaA box perfectly matched the optimal consensus TTATCCACA in all *M. feriruminatoris* strains, whereas only imperfect DnaA boxes were predicted in *M. mycoides *-cluster species. Downstream of the *dnaA* gene, one perfect DnaA box was found in *M. feriruminatoris* and in all other strains from the *M. mycoides* cluster. This remarkable feature suggests that the replication initiation process might be accelerated by enhanced binding of DnaA to the *M. feriruminatoris oriC* region. Further experiments based on DnaA box mutagenesis will be necessary to test this hypothesis.

In conclusion, this comparative genomics study confirmed the genomic boundaries of the *M. feriruminatoris* species. The current set of isolates available comes primarily from two locations in Europe, with the exception of one strain isolated in the US. New isolates might reveal a larger genetic diversity, but it should be stressed that despite having a very different geographical origin and year of isolation, the US strain 8756–13 was phylogenetically close to some recent European strains and presented a similar gene content. This is in accordance with the closed pan-genome that was extrapolated from the *M. feriruminatoris* strains included in this study. The intraspecies variability is limited and mainly due to mobile elements such as IS, MICEs, and even a plasmid detected in one isolate only. The *M. feriruminatoris* species is very closely related to the *M. mycoides* cluster, as demonstrated by its phylogenetic positioning but also by its gene content and genome organization as well as several typical characteristics (plasmid, lipoprotein repertoire, production of galactan and glucan, etc.). Therefore, we propose to extend the perimeter of the *M. mycoides* cluster to include the *M. feriruminatoris* species. The evolution of *M. feriruminatoris* is associated with both losses and gains of genes, but further studies will be necessary to determine if these could explain the host specificity of *M. feriruminatoris* to wild *Caprinae*. Indeed, no spillover to domesticated ruminants has been detected so far. Finally, yet importantly, further work is needed to assess whether the specific organization and structure of the DnaA boxes around the *oriC* of the *M. feriruminatoris* genomes could explain its growth characteristics. The recent development of highly efficient in-yeast genome engineering methods and genome transplantation protocols for *M. feriruminatoris* [29] now opens up ways to tackle the questions raised by our study.

## Supplementary Data

Supplementary material 1

Supplementary material 2

Supplementary material 3

Supplementary material 4

## References

[1] Brown DR, May M, Bradbury JM, Johansson K-E (2018). Bergey’s Manual of Systematics of Archaea and Bacteria.

[2] Noll LW, Highland MA, Hamill VA, Tsui WNT, Porter EP (2022). Development of a real-time PCR assay for detection and differentiation of *Mycoplasma ovipneumoniae* and a novel respiratory-associated *Mycoplasma* species in domestic sheep and goats. Transbound Emerg Dis.

[3] Spergser J, DeSoye P, Ruppitsch W, Cabal Rosel A, Dinhopl N (2022). *Mycoplasma tauri* sp. nov. isolated from the bovine genital tract. Syst Appl Microbiol.

[4] Volokhov DV, Furtak VA, Blom J, Zagorodnyaya TA, Gao Y (2022). *Mycoplasma miroungirhinis* sp. nov. and *Mycoplasma miroungigenitalium* sp. nov., isolated from northern elephant seals (*Mirounga angustirostris*), *Mycoplasma phocoenae* sp. nov., isolated from harbour porpoise (*Phocoena phocoena*), and *Mycoplasma phocoeninasale* sp. nov., isolated from harbour porpoise and California sea lions (*Zalophus californianus*). Int J Syst Evol Microbiol.

[5] Brown DR, May M, Bradbury JM, Balish MF, Calcutt MJ (2018). Bergey’s Manual of Systematics of Archaea and Bacteria.

[6] Gasparich GE, Whitcomb RF, Dodge D, French FE, Glass J (2004). The genus *Spiroplasma* and its non-helical descendants: phylogenetic classification, correlation with phenotype and roots of the *Mycoplasma mycoides* clade. Int J Syst Evol Microbiol.

[7] Lo W-S, Gasparich GE, Kuo C-H (2018). Convergent evolution among ruminant-pathogenic *Mycoplasma* involved extensive gene content changes. Genome Biol Evol.

[8] Sirand-Pugnet P, Lartigue C, Marenda M, Jacob D, Barré A (2007). Being pathogenic, plastic, and sexual while living with a nearly minimal bacterial genome. PLoS Genet.

[9] Cottew GS, Breard A, DaMassa AJ, Ernø H, Leach RH (1987). Taxonomy of the *Mycoplasma mycoides* cluster. Isr J Med Sci.

[10] Manso-Silván L, Perrier X, Thiaucourt F (2007). Phylogeny of the *Mycoplasma mycoides* cluster based on analysis of five conserved protein-coding sequences and possible implications for the taxonomy of the group. Int J Syst Evol Microbiol.

[11] Fischer A, Shapiro B, Muriuki C, Heller M, Schnee C (2012). The origin of the “mycoplasma mycoides cluster” coincides with domestication of ruminants. PLoS One.

[12] Manso-Silván L, Vilei EM, Sachse K, Djordjevic SP, Thiaucourt F (2009). *Mycoplasma leachii* sp. nov. as a new species designation for *Mycoplasma* sp. bovine group 7 of Leach, and reclassification of *Mycoplasma mycoides* subsp. mycoides LC as a serovar of *Mycoplasma mycoides* subsp. *capri*. Int J Syst Evol Microbiol.

[13] Tardy F, Maigre L, Poumarat F, Citti C (2009). Identification and distribution of genetic markers in three closely related taxa of the *Mycoplasma mycoides* cluster: refining the relative position and boundaries of the *Mycoplasma* sp. bovine group 7 taxon (*Mycoplasma leachii*). Microbiology.

[14] Jores J, Fischer A, Sirand-Pugnet P, Thomann A, Liebler-Tenorio EM (2013). *Mycoplasma feriruminatoris* sp. nov., a fast growing *Mycoplasma* species isolated from wild *Caprinae*. Syst Appl Microbiol.

[15] Ambroset C, Pau-Roblot C, Game Y, Gaurivaud P, Tardy F (2017). Identification and characterization of *Mycoplasma feriruminatoris* sp. nov. strains isolated from Alpine ibex: a 4th species in the *Mycoplasma mycoides* cluster hosted by non-domesticated ruminants?. Front Microbiol.

[16] Tardy F, Baranowski E, Nouvel L-X, Mick V, Manso-Silvàn L (2012). Emergence of atypical *Mycoplasma agalactiae* strains harboring a new prophage and associated with an alpine wild ungulate mortality episode. Appl Environ Microbiol.

[17] Fischer A, Santana-Cruz I, Giglio M, Nadendla S, Drabek E (2013). Genome sequence of *Mycoplasma feriruminatoris* sp. nov., a fast-growing *Mycoplasma* species. Genome Announc.

[18] Jores J, Ma L, Ssajjakambwe P, Schieck E, Liljander A (2019). Removal of a subset of non-essential genes fully attenuates a highly virulent *Mycoplasma* strain. Front Microbiol.

[19] Hutchison CA, Chuang R-Y, Noskov VN, Assad-Garcia N, Deerinck TJ (2016). Design and synthesis of a minimal bacterial genome. Science.

[20] Lartigue C, Glass JI, Alperovich N, Pieper R, Parmar PP (2007). Genome transplantation in bacteria: changing one species to another. Science.

[21] March JB, Clark J, Brodlie M (2000). Characterization of strains of *Mycoplasma mycoides* subsp. *mycoides* small colony type isolated from recent outbreaks of contagious bovine pleuropneumonia in Botswana and Tanzania: evidence for a new biotype. J Clin Microbiol.

[22] Jores J, Baldwin C, Blanchard A, Browning GF, Colston A (2020). Contagious bovine and caprine pleuropneumonia: a research community’s recommendations for the development of better vaccines. NPJ Vaccines.

[23] Schumacher M, Nicholson P, Stoffel MH, Chandran S, D’Mello A (2019). Evidence for the cytoplasmic localization of the L-α-glycerophosphate oxidase in members of the “*Mycoplasma mycoides* cluster.”. Front Microbiol.

[24] Szczepanek SM, Boccaccio M, Pflaum K, Liao X, Geary SJ (2014). Hydrogen peroxide production from glycerol metabolism is dispensable for virulence of *Mycoplasma gallisepticum* in the tracheas of chickens. Infect Immun.

[25] Vilei EM, Frey J (2001). Genetic and biochemical characterization of glycerol uptake in *Mycoplasma mycoides* subsp. *mycoides* SC: its impact on H(2)O(2) production and virulence. Clin Diagn Lab Immunol.

[26] Jores J, Schieck E, Liljander A, Sacchini F, Posthaus H (2019). In vivo role of capsular polysaccharide in *Mycoplasma mycoides*. J Infect Dis.

[27] Gaurivaud P, Baranowski E, Pau-Roblot C, Sagné E, Citti C (2016). *Mycoplasma agalactiae* secretion of β-(1→6)-glucan, a rare polysaccharide in prokaryotes, is governed by high-frequency phase variation. Appl Environ Microbiol.

[28] Gaurivaud P, Lakhdar L, Le Grand D, Poumarat F, Tardy F (2014). Comparison of in vivo and in vitro properties of capsulated and noncapsulated variants of *Mycoplasma mycoides* subsp. *mycoides* strain Afadé: a potential new insight into the biology of contagious bovine pleuropneumonia. FEMS Microbiol Lett.

[29] Talenton V, Baby V, Gourgues G, Mouden C, Claverol S (2022). Genome engineering of the fast growing *Mycoplasma feriruminatoris*, towards a functional chassis for veterinary vaccines. ACS Synth Biol.

[30] Poumarat F, Jarrige N, Tardy F (2014). Purpose and overview of results of the vigimyc network for the epidemiological surveillance of *Mycoplasmoses* in ruminants in France. Euroreference.

[31] Labroussaa F, Thomann A, Nicholson P, Falquet L, Jores J (2020). Complete genome sequence of *Mycoplasma feriruminatoris* Strain IVB14/OD_0535, Isolated from an Alpine Ibex in a Swiss Zoo. Microbiol Resour Announc.

[32] Poumarat F, Longchambon D, Martel JL (1992). Application of dot immunobinding on membrane filtration (MF dot) to the study of relationships within “*M. mycoides* cluster” and within “glucose and arginine-negative cluster” of ruminant mycoplasmas. Vet Microbiol.

[33] Chen WP, Kuo TT (1993). A simple and rapid method for the preparation of gram-negative bacterial genomic DNA. Nucleic Acids Res.

[34] Bolger AM, Lohse M, Usadel B (2014). Trimmomatic: a flexible trimmer for Illumina sequence data. Bioinformatics.

[35] Koren S, Walenz BP, Berlin K, Miller JR, Bergman NH (2017). Canu: scalable and accurate long-read assembly via adaptive *k*-mer weighting and repeat separation. Genome Res.

[36] Li H, Durbin R (2009). Fast and accurate short read alignment with burrows–wheeler transform. Bioinformatics.

[37] Walker BJ, Abeel T, Shea T, Priest M, Abouelliel A (2014). Pilon: an integrated tool for comprehensive microbial variant detection and genome assembly improvement. PLoS One.

[38] McKenna A, Hanna M, Banks E, Sivachenko A, Cibulskis K (2010). The genome analysis toolkit: a mapreduce framework for analyzing next-generation DNA sequencing data. Genome Res.

[39] Li H, Handsaker B, Wysoker A, Fennell T, Ruan J (2009). The sequence alignment/map format and SAMtools. Bioinformatics.

[40] Seemann T (2014). Prokka: rapid prokaryotic genome annotation. Bioinformatics.

[41] Contreras-Moreira B, Vinuesa P (2013). GET_HOMOLOGUES, a versatile software package for scalable and robust microbial pangenome analysis. Appl Environ Microbiol.

[42] Kristensen DM, Kannan L, Coleman MK, Wolf YI, Sorokin A (2010). A low-polynomial algorithm for assembling clusters of orthologous groups from intergenomic symmetric best matches. Bioinformatics.

[43] Darling ACE, Mau B, Blattner FR, Perna NT (2004). Mauve: multiple alignment of conserved genomic sequence with rearrangements. Genome Res.

[44] Darling AE, Mau B, Perna NT, Stajich JE (2010). Progressivemauve: multiple genome alignment with gene gain, loss and rearrangement. PLoS ONE.

[45] Popendorf K, Tsuyoshi H, Osana Y, Sakakibara Y (2010). Murasaki: a fast, parallelizable algorithm to find anchors from multiple genomes. PLoS One.

[46] Citti C, Dordet-Frisoni E, Nouvel LX, Kuo CH, Baranowski E (2018). Horizontal gene transfers in *Mycoplasmas* (*Mollicutes*). Curr Issues Mol Biol.

[47] Siguier P, Perochon J, Lestrade L, Mahillon J, Chandler M (2006). ISfinder: the reference centre for bacterial insertion sequences. Nucleic Acids Res.

[48] Marchler-Bauer A, Bryant SH (2004). CD-Search: protein domain annotations on the fly. Nucleic Acids Res.

[49] Bailey TL, Boden M, Buske FA, Frith M, Grant CE (2009). MEME SUITE: tools for motif discovery and searching. Nucleic Acids Res.

[50] Teufel F, Almagro Armenteros JJ, Johansen AR, Gíslason MH, Pihl SI (2022). SignalP 6.0 predicts all five types of signal peptides using protein language models. Nat Biotechnol.

[51] Hallgren J, Tsirigos KD, Pedersen MD, Almagro Armenteros JJ, Marcatili P (2022). DeepTMHMM predicts alpha and beta transmembrane proteins using deep neural networks. Bioinformatics.

[52] Schieck E, Lartigue C, Frey J, Vozza N, Hegermann J (2016). Galactofuranose in *Mycoplasma mycoides* is important for membrane integrity and conceals adhesins but does not contribute to serum resistance. Mol Microbiol.

[53] Krogh A, Larsson B, von Heijne G, Sonnhammer EL (2001). Predicting transmembrane protein topology with a hidden Markov model: application to complete genomes. J Mol Biol.

[54] Sievers F, Wilm A, Dineen D, Gibson TJ, Karplus K (2011). Fast, scalable generation of high-quality protein multiple sequence alignments using clustal omega. Mol Syst Biol.

[55] Talavera G, Castresana J (2007). Improvement of phylogenies after removing divergent and ambiguously aligned blocks from protein sequence alignments. Syst Biol.

[56] Darriba D, Taboada GL, Doallo R, Posada D (2011). ProtTest 3: fast selection of best-fit models of protein evolution. Bioinformatics.

[57] Adachi J, Waddell PJ, Martin W, Hasegawa M (2000). Plastid genome phylogeny and a model of amino acid substitution for proteins encoded by chloroplast DNA. J Mol Evol.

[58] Stamatakis A (2014). RAxML version 8: a tool for phylogenetic analysis and post-analysis of large phylogenies. Bioinformatics.

[59] Csurös M (2010). Count: evolutionary analysis of phylogenetic profiles with parsimony and likelihood. Bioinformatics.

[60] Moldovan MA, Gelfand MS (2018). Pangenomic definition of prokaryotic species and the phylogenetic structure of *Prochlorococcus* spp. Front Microbiol.

[61] Arfi Y, Minder L, Di Primo C, Le Roy A, Ebel C (2016). MIB-MIP is a mycoplasma system that captures and cleaves immunoglobulin G. Proc Natl Acad Sci USA.

[62] Frey J, Cheng X, Kuhnert P, Nicolet J (1995). Identification and characterization of IS1296 in *Mycoplasma mycoides* subsp. *mycoides* SC and presence in related mycoplasmas. Gene.

[63] Thiaucourt F, Manso-Silvan L, Salah W, Barbe V, Vacherie B (2011). *Mycoplasma mycoides*, from “mycoides small colony” to “capri”. A microevolutionary perspective. BMC Genomics.

[64] Hill V, Akarsu H, Barbarroja RS, Cippà VL, Kuhnert P (2021). Minimalistic mycoplasmas harbor different functional toxin-antitoxin systems. PLoS Genet.

[65] Gourgues G, Barré A, Beaudoing E, Weber J, Magdelenat G (2016). Complete genome sequence of *Mycoplasma mycoides* subsp. *mycoides* T1/44, a vaccine strain against contagious bovine pleuropneumonia. Genome Announc.

[66] Breton M, Tardy F, Dordet-Frisoni E, Sagne E, Mick V (2012). Distribution and diversity of mycoplasma plasmids: lessons from cryptic genetic elements. BMC Microbiol.

[67] Moscoso M, del Solar G, Espinosa M (1995). Specific nicking-closing activity of the initiator of replication protein RepB of plasmid pMV158 on supercoiled or single-stranded DNA. J Biol Chem.

[68] Westberg J, Persson A, Holmberg A, Goesmann A, Lundeberg J (2004). The genome sequence of *Mycoplasma mycoides* subsp. *mycoides* SC type strain PG1T, the causative agent of contagious bovine pleuropneumonia (CBPP). Genome Res.

[69] Bischof DF, Vilei EM, Frey J (2006). Genomic differences between type strain PG1 and field strains of *Mycoplasma mycoides* subsp. *mycoides* small-colony type. Genomics.

[70] Bertin C, Pau-Roblot C, Courtois J, Manso-Silván L, Tardy F (2015). Highly dynamic genomic loci drive the synthesis of two types of capsular or secreted polysaccharides within the *Mycoplasma mycoides* cluster. Appl Environ Microbiol.

[71] Kamminga T, Koehorst JJ, Vermeij P, Slagman S-J, Martins Dos Santos VAP (2017). Persistence of functional protein domains in Mycoplasma species and their role in host specificity and synthetic minimal life. Front Cell Infect Microbiol.

[72] Röske K, Foecking MF, Yooseph S, Glass JI, Calcutt MJ (2010). A versatile palindromic amphipathic repeat coding sequence horizontally distributed among diverse bacterial and eucaryotic microbes. BMC Genomics.

[73] Willenbrock H, Hallin PF, Wassenaar TM, Ussery DW (2007). Characterization of probiotic *Escherichia coli* isolates with a novel pan-genome microarray. Genome Biol.

[74] Tettelin H, Masignani V, Cieslewicz MJ, Donati C, Medini D (2005). Genome analysis of multiple pathogenic isolates of *Streptococcus agalactiae*: implications for the microbial “pan-genome.”. Proc Natl Acad Sci U S A.

